# Supplementary material for: PR3 levels are impaired in plasma and PBMCs from Arabs with cardiovascular diseases
Source: PLoS One. 2020 Jan 14;15(1):e0227606. doi: 10.1371/journal.pone.0227606 (PMC6959567; doi:10.1371/journal.pone.0227606)
Supplement: S1 Table — (DOCX) [file pone.0227606.s001.docx]

**S1 Table:** Primer sequences used for real time PCR to analyze gene expression status of selected genes

| *Genes* | Forward primers | Reverse primers |
| --- | --- | --- |
| *ARHGAP30* | 5'-GCTTCCTCTTCAACAGCCAC-3' | 5'-TCCTTTCTGCCGAGACTTCA-3' |
| *RPA2* | 5'-CGTTTGTGGTGCCAGAGAAA-3' | 5'-CGCCCTCTTGCTAAAACCTC-3' |
| *AMPHL* | 5'-TGTAGGAAGAGCAGTGTGGG-3' | 5'-TATAGGAAGTGGCTGGCACC-3' |
| *ERP44* | 5'-GAAGTGAGGGTGTGAGAGGC-3' | 5'-AAGGATAGGAAGACGGCAAGG-3' |
| *PPM1G* | 5'-GGATCACGAGGTCAGGAGTT-3' | 5'-TCCCAGGTTCAAGCGATTCT-3' |
| *TXNDC17* | 5'-ACCACCTTACCCGGATGATC-3' | 5'-ACCAAATCCCAGTCCCGATT-3' |
| *KIF5B* | 5'-GAAGAAGGTGGCCACTCTCC-3' | 5'-GGCAGCCGTTAACCCTAATG-3' |
| *CPNE1* | 5'-AGCTTTCCCCGGTAAGACTC-3' | 5'-GTCCTCCTTGCATCCTCTCA-3' |
| *NAPIL4* | 5'-AAGGTCAAGGCGCTTTGTTC-3' | 5'-CTGGGCTAGCGGGAGATTT-3' |
| *EIF2A* | 5'-CCTGGGGCAATGGAGAAAAG-3' | 5'-AGGTATTTTGTGTAACGAGAGGT-3' |
| *PRTN3 (PR3)* | 5'-AGACTCCTTCGTGATCTGGG-3' | 5'-TTCTGTCCAAAGATCCGCCT-3' |
| *DEFA1* | 5'-AGAATACCAGCGTGCATTGC-3' | 5'-TGAGCAGAAGGTACAGGAGT-3' |
| *CLG4B (MMP9)* | 5'-CGCTATGGTTACACTCGGGT-3' | 5'-CTATCCAGCTCACCGGTCTC-3' |
| *ANX3* | 5'-GGTGATCTCTCTGGCCACTT-3' | 5'-AGTCCACCCATCACAAGTGT-3' |
| *OXCT* | 5'-GGGTTTCCTTCGCAGTCG-3' | 5'-AGGAGAGGAGTTTGAGAGCC-3' |
| *SPTB* | 5'-CGCCAAGAAGAAGCATGAGG-3' | 5'-AGCATGTCCTGGAAGAGCTT-3' |
| *FLOT2* | 5'-CTGCTGTTGTCCGGGTCT-3' | 5'-CCCTCACCTGAAACCACCA-3' |
| *SPTA* | 5'-TAAGGGCAAGGCAAGAGTCC-3' | 5'-GGTGGAAGTAGCTGAGGGTTT-3' |
| *A4* | 5'-GCTCTGGGGACTCTGGTTTA-3' | 5'-GCTTTTCTTTGCTGCACTGC-3' |
| *GAPDH* | 5’-AGGGCTGCTTTTAACTCTGGT-3’ | 5’-CCCCACTTGATTTTGGAGGGA-3’ |
