# Supplementary material for: PR3 levels are impaired in plasma and PBMCs from Arabs with cardiovascular diseases
Source: PLoS One. 2020 Jan 14;15(1):e0227606. doi: 10.1371/journal.pone.0227606 (PMC6959567; doi:10.1371/journal.pone.0227606)
Supplement: S2 Table — (DOCX) [file pone.0227606.s002.docx]

**S2Table****: Characteristics of subjects used in proteomics profiling and RT-PCR validation**

| Parameter | Controls | Cases | P-value |
| --- | --- | --- | --- |
| Total subjects  (Male/Female) | 10  (5 / 5) | 10  (5 / 5) |  |
| Age (years) | 55.5 ± 7.8 | 56.3 ± 7.1 | 0.736 |
| BMI | 31.1 ± 5.2 | 31.6 ± 5.5 | 0.735 |
| Waist (cm) | 100.3 ± 9.6 | 99.8 ± 11.4 | 0.693 |
| Hip (cm) | 110.7 ± 8.6 | 109.8 ± 9.3 | 0.562 |
| SBP (mmHg) | 129.5 ± 17.2 | 132.1 ± 19.7 | 0.271 |
| DBP (mmHg) | 79.1 ± 8.9 | 78.4 ± 11.7 | 0.164 |
| FBG (mmol/l) | 5.1 ± 0.4 | 5.2 ± 0.5 | 0.273 |
| HbA1c (%) | 5.4 ± 0.3 | 5.5 ± 0.5 | 0.219 |
| TC (mmol/l) | 5.3 ± 0.9 | 4.9 ± 1.1 | **0.037** |
| TG (mmol/l) | 1.4 ± 0.7 | 1.6 ± 1.2 | 0.172 |
| HDL (mmol/l) | 1.2 ± 0.3 | 1.1 ± 0.4 | 0.184 |
| LDL (mmol/l) | 3.4 ± 0.7 | 3.1 ± 0.8 | 0.087 |
| oxLDL (ug/l) | 19.8 (5.4-55.8) | 17.1 (3.4-43.1) | 0.073 |
| hsCRP (ug/l) | 2.1 (0.2-8.9) | 3.0 (1.1-9.7) | **0.041** |

*Data are presented as mean ± SD. Body mass index (BMI), Systolic blood pressure (SBP), Diastolic blood pressure (DBP), High density lipoprotein (HDL), Low density lipoprotein (LDL), Triglycerides (TG). Non-parameteric Mann-Whitney test was used to determine significance of difference in means between case and control groups.*
