# Supplementary material for: PR3 levels are impaired in plasma and PBMCs from Arabs with cardiovascular diseases
Source: PLoS One. 2020 Jan 14;15(1):e0227606. doi: 10.1371/journal.pone.0227606 (PMC6959567; doi:10.1371/journal.pone.0227606)
Supplement: Attachment — Submitted filename: Khadir-PR3_Reply to Reviewers comments.docx [file pone.0227606.s004.docx]

**Reply to Reviewer’ comments**

We would like first, to thank the reviewers for their overall positive feedback on our manuscript. We also thank them for the valuable suggestions they made to enhance the quality of our draft.

In our reply, we have addressed all the queries that were raised and changes, whenever applicable, were incorporated in the revised manuscript and shown in blue color. Please find below our point-by point responses to the reviewer’ comments.

**Reviewers' comments:**
Reviewer's Responses to Questions

**Comments to the Author**
1. Is the manuscript technically sound, and do the data support the conclusions?
The manuscript must describe a technically sound piece of scientific research with data that supports the conclusions. Experiments must have been conducted rigorously, with appropriate controls, replication, and sample sizes. The conclusions must be drawn appropriately based on the data presented.
Reviewer #1: Yes
Reviewer #2: Partly
Reviewer #3: Yes

2. Has the statistical analysis been performed appropriately and rigorously?
Reviewer #1: I Don't Know
Reviewer #2: Yes
Reviewer #3: Yes

3. Have the authors made all data underlying the findings in their manuscript fully available?
The [PLOS Data policy](http://www.plosone.org/static/policies.action#sharing) requires authors to make all data underlying the findings described in their manuscript fully available without restriction, with rare exception (please refer to the Data Availability Statement in the manuscript PDF file). The data should be provided as part of the manuscript or its supporting information or deposited to a public repository. For example, in addition to summary statistics, the data points behind means, medians and variance measures should be available. If there are restrictions on publicly sharing data—e.g. participant privacy or use of data from a third party—those must be specified.
Reviewer #1: Yes
Reviewer #2: Yes
Reviewer #3: Yes

4. Is the manuscript presented in an intelligible fashion and written in standard English?

PLOS ONE does not copyedit accepted manuscripts, so the language in submitted articles must be clear, correct, and unambiguous. Any typographical or grammatical errors should be corrected at revision, so please note any specific errors here.
Reviewer #1: Yes
Reviewer #2: Yes
Reviewer #3: Yes

**5. Review Comments to the Author**

Please use the space provided to explain your answers to the questions above. You may also include additional comments for the author, including concerns about dual publication, research ethics, or publication ethics. (Please upload your review as an attachment if it exceeds 20,000 characters)

**Reviewer #1:** In the present MS, Authors Abdelkrim Khadir and collaborators, investigated cardiovascular disease (CVD) biomarkers in circulating blood cells (PBMCs) and plasma from Arab obese subjects with and without CVD and diabetes.
By using proteomics tools Authors identified Proteinase-3 (PR3) as a putative independently biomarker associated with CVD in the plasma and PBMCs.
This work deals about a subject of great interest and arises from a very capable group and used valuable techniques.
That said I have few points that should be addressed by the Authors:

1- I have a little remark concerning the term "Arabs" in the title of the MS: "PR-3 levels are impaired in plasma and PBMCs from Arabs with cardiovascular diseases". I understood the study were performed on Human adults living in Kuwait. As inhabitants of Kuwait are called Kuwaitis, I thought the term "Kuwaitis" would be more appropriate. Please give me your opinion on this.

We thank the reviewer for this comment. In our cohort we have enrolled both Kuwaiti nationals and non-Kuwaiti Arabs living in Kuwait from other nationalities (Palestinians, Egyptians, Syrians…). Hence, we have used the term “Arabs” and not “Kuwaiti”.

2- The abstract of the MS is not very clear-cut and therefore could be improved. To my point of view, it lacks a logical progression. It should be more informative in terms of results.

We agree with the reviewer on this point. Thus, as suggested, we have changed the abstract and included more results and technical details with a logical progression. All modifications are highlighted in blue in the abstract of the revised version.

Aims of the Authors is to identify CVD biomarkers in blood cells and plasma from Arab obese subjects with and without CVD and diabetes. Then, how come there are only two groups “Human adults with CVD (n = 208) and controls (n = 152)”?

We thank the reviewer for this comment. In our cohort we have enrolled 2 adult groups with or without CVD as detailed in Table 3. Nevertheless, some individuals from each group have diabetes which is already a risk factor for CVD. Thus, in Table 5 we have separated the cohort and analyzed them according to their diabetes status (controls without diabetes, controls with diabetes, CVD without diabetes, CVD with diabetes). In those 4 groups, we have measured and reported the plasma levels of our biomarkers. In the PBMCs, and as we have mentioned the first paragraph of the Results section, we have used only limited set of representative samples of non-diabetic human adults with reported CVD-related events and their matched healthy controls. Another set of six subjects from each group were used later for expression validation using mRNA and real-time PCR (RT-PCR). Characteristics of the subjects are displayed in Table S2.

For further avoid any confusion, we have removed the word diabetes from out aim (in the abstract) and now the sentence reads as: “*The aim of the present study was to identify novel CVD candidate markers in circulating blood cells and plasma from Arab obese subjects with and without CVD.”*

Authors employed “a shotgun proteomic profiling approach on PBMCs isolated from a subset of the subjects, and differentially expressed proteins selected between the two groups were validated at the mRNA level using RT-PCR.” What subset of subjects did Authors use? How many differentially expressed did the Authors identified? Why validation was made at the mRNA level and not the protein level?

We thank the reviewer for these comments. In our study, we have used, as detailed in the first paragraph of the Results section, a set of 4 representative samples of non-diabetic human adults with reported CVD-related events and 4 from their matched healthy controls. Another set of six subjects from each group were used later for expression validation using mRNA and real-time PCR (RT-PCR). Characteristics of the subjects are displayed in Table S2 and are comparable to the whole population (summarized in Table 3).

- The number of differentially expressed proteins were also detailed in the results section and Table Sxxx.
- The validation of protein expression was performed using mRNA because it an orthogonal validation where we use a different type of biological material and thus it avoids potential experimental biases. We could use Western blots to quantify protein expression, but as both proteomics and Western Blot use proteins, any experimental bias due to protein extraction or processing will affect similarly both analyses.

To clarify further these points, we have changed some statements in the Abstract section and added various technical details (highlighted in blue).

Then after, in the abstract Authors wrote about Proteinase-3 (PR3), Annexin-A3 (ANX3), Defensin (DEFA1), and Matrix Metalloproteinase9 (MMP9), which plasma levels were measured by ELISA. What is the link with was written before and after this statement?

We thank the reviewer for this kind comment. The 4 listed proteins are the markers differentially expressed between the two groups and which we have chosen from the proteomics profiling to validate in the whole cohort based on their role and interconnection in metabolic diseases.

As suggested, we have added some statements in the abstract to make this clear.

Just after, they wrote "We identified 47 dysregulated proteins", again I do understand the link and progression in the abstract.
Some clarifications and statements were added to the abstract section as mentioned above.

In the abstract, sentence starting with "Despite the decreases..." is very long and complex. It should be rephrased and simplified.

As suggested by the reviewer, this statement was rephrased and simplified. Please see the Abstract in revised version.

3- Introduction is very readable and clear. End of the introduction nicely itemize the different objectives of the work (pls refer to this when rephrased your abstract).

We thank the reviewer for this kind comment. As suggested, we have used some of these statement for the revised abstract.

4- page 11, a reference should be included for the "Declaration of Helsinki" statement.
page 11 lane 99 " low-density Lipoprotein" no capital for lipoprotein

We agree with the reviewer on these points.

As suggested, we have added a reference to the “Declaration of Helsinki” with the most updated amendments (64th WMA General Assembly, Fortaleza, Brazil, October 2013). We have also corrected the capital spelling in lipoprotein.

5- method section page 11, Authors should develop methods used for blood parameter measurements, TG LDL ...How did Authors measured oxidized LDL?

We apologize for missing these experimental details in our manuscript. As suggested by the reviewer, details on blood parameters, OxLDL and hsCRP were added in the Material and Methods section and highlighted in blue. Please see page 5 of our revised version.

6- page 31 table 3. To my point of view, term "gender" should be replace by "sex" which is used in Science and corresponds to the genetic equipment (22 chr + XX or 22 ch +XY)

We agree with the reviewer on this point related to the dilemma of reporting Sex or Gender in science and research. Hence, we have substituted “gender’ by “sex” in all the manuscript (8 times).

7- page 36 legend of figure 1 should be developed. What is the signification of the colors of the lines, their width ...

As suggested by the reviewer, we have developed further the legend and we added the color codes for the figure as per the software specifications.

8- Page 14 lane 300, 302 and 303 Authors wrote CAD. I did not find the meaning of this acronym.

We apologize for not defining the acronym. We have now defined it in the revised version as: “coronary artery disease (CAD)”.

9- I think Authors should develop a bit more about biomarkers they identified (PR3, DEFA1, MMP9 and ANX3) in terms of tissue origin, protein localization and role in the body.

We agree with the reviewer on this point and we limited the details in our previous manuscript version to keep it concise. As suggested by the reviewer, we have added more details on these markers and the modifications are highlighted in blue on page 13.

The paragraph reads now as:

*“MMP9 proteins belong to a family of metalloproteases that degrade extracellular matrix (ECM) and are involved in normal tissue remodeling; however, their induction is associated with several pathological conditions including chronic inflammation [14]. In humans but not rodents, neutrophil MMP9 is covalently linked with lipocalin and hence, protected from proteolysis while in various pathologies MMP9 are localized in the nucleus [15]. MMP9 proteins are also implicated in several stages of atherosclerosis involving leukocyte adhesion, cell migration, and matrix degradation [16]. Studies have reported elevated levels of MMP9 mainly in patients and animals with acute myocardial infarction (AMI) and acute coronary syndrome (ACS) [17-19]. DEFA1 is a member of the Defensin neutrophil peptides family, known to be cysteine-rich positively charged, that are secreted into circulation [20]. It was also reported to be stored in granules [21]. It binds to endothelial cells in vitro and accumulates in the intima of atherosclerotic vessels [20]. Recently, DEFA1 expression levels have been reported to be associated with coronary heart disease (CHD) in hyperlipidemic patients [22]. ANX3, a member of the calcium-dependent phospholipid-binding protein family, plays a role in the regulation of cellular growth and in signal transduction pathways [23]. It is also associated with cytoplasmic granules and translocates to the plasma membrane in activated blood cells [24]. ANX3 expression increases in post-ischemic brains [25].*

*On the other hand, PR3 is a neutrophil serine protease, mainly stored in intracellular granules, that degrades ECM [26]. PR3 is also expressed on endothelial cells [27] and was reported to promote inflammatory response, induce vascular damage, and trigger endothelial cell apoptosis, particularly in Chronic Obstructive Pulmonary Disease (COPD) [28]. Notably, in the context of CVDs, PR3 is primarily reported to have deleterious effects in the pathogenesis of vascular inflammation such as vasculitis in Wegener’s granulomatosis, and potentially in the prognosis for patients post-AMI [29]. However, a significant role of PR3 in disease development has emerged recently not only in COPD but also in other chronic inflammatory conditions, where PR3 is considered not only as an autoantigen but also for its involvement in the modulation of inflammatory pathways and cellular signaling [28].”*

10- Page 15 lane 341 Authors wrote “when analyzing a larger cohort of Arab ethnic groups (ref).”

We apologize for this mistake. In fact, we were discussing and referring to our current results and not another study. Thus, we have removed the mention “ref” and we adjust the text accordingly.

The statement now is: “*In contrast to the decreased expression levels of the four genes and proteins in the PBMCs of a small set of subjects, no differences were observed in the DEFA1 and ANX3 plasma levels when analyzing a larger cohort of subjects in the present study*.”

11- Page 16 lane 358 Authors wrote “CHD biomarker CHD”

We apologize for this typo mistake. We have removed the second CHD word from the text.

12- Authors included a limitation section at the end of the MS. If they write the present work is mostly descriptive, Authors could also develop on researches needed to get insight into a putative causal role of PR3 in CVD.

We thank the reviewer for this interesting suggestion to improve our manuscript despite we have mentioned some perspectives in the conclusion section. Accordingly, we have suggested some research directions which will help in addressing the putative causal role of PR3 in CVDs.

The following statement was added at the end of the limitation paragraph: “*Further investigations are warranted to address the putative causal role of PR3 in CVDs through prospective larger cohort studies including subjects at risk for CVD and assessing not only the PR3 expression levels but also its activity and autoantibodies. Knockout or upregulated gene expression of PR3 in animal models would also elucidate the contribution of PR3 to CVD onset and progression.*”

**Reviewer #2:** Review on the manuscript entitled: “PR-3 levels are impaired in plasma and PBMCs from Arabs with cardiovascular diseases” by Khadir A et al.
This article’s aim is to identify novel CVD markers in circulating blood cells and plasma from Arab obese subjects with and without CVD and diabetes. A number of biochemical parameters (PR3, ANX3, DEFA1 and MMP9) were analyzed using a shotgun proteomic profiling approach on PBMCs isolated from a subset of subjects.

This was quite an extensive study that was well designed and equally well written. That being said there are some aspects that the authors need to attend to.

• In the abstract it is not mentioned whether the study is cross-sectional or longitudinal, though this is mentioned later on in the discussion.

We thank the reviewer for this comment. We have now mentioned the cross-sectional type of our study in the revised abstract.

• Furthermore, the methodology should clarify how the sampling process was done, stating both the inclusion and exclusion criteria not only in the abstract but also for the manuscript.

We thank the reviewer for this critical point. In fact, we have previously reported the details on the sampling process of subjects and their inclusion and exclusion criteria. To avoid redundancy, we have briefly mentioned in our revised manuscript the process and added the adequate reference for further details.

Thus, the statement now is: “*The current study included 208 Arab adults with confirmed CVD-related events (cases) and 152 healthy controls with no reported CVD-related events. Those subjects and their samples were extracted from the Kuwait Diabetes Epidemiology Program (KDEP) that was conducted between June 2011 and August 2012 at the Dasman Diabetes Institute (DDI) to estimate the prevalence of non-communicable diseases among the resident population of Kuwait [10].*”

• At the same time, the authors do mention that the individuals were “apparently” healthy. Who was determining the health status of the participants in the study? And this comes back to the sampling method and/or exclusion vs. inclusion criteria.

We thank the reviewer for this comment. We have removed the word “apparently” from our material and Methods section, to avoid any confusion, as the subjects were thoroughly screened and categorized by our clinicians. Furthermore, the control subjects had no reported CVD or other major diseases both through the questionnaires and their medical health records. As mentioned in the above point, we have referred the readers for the appropriate reference for further details.

• It is stated both in the abstract and the introduction that the shotgun proteomic profiling techniques were used. However, the methods section does not mention shotgun profiling as such though it is described. It would be helpful to mention it as well.

We thank the reviewer for this comment. Just for clarification, Shotgun is a proteomic approach where proteins are digested without separation and then analyzed throw liquid chromatography separation and mass spectrometry, in opposition with the analysis of intact proteins (Top-down approach). As suggested by the reviewer, we have mentioned again the shotgun approach in Materiel and Methods section.

• The discussion section was well executed and answered most of the questions I had, in particular the limitations of the study. One in particular on the different levels of PR3 in the circulation and that of the mRNA and protein expressed.

We thank the reviewer for this positive and motivating comment

• There are some minor typos in the paper…
Line 93
Line 163

Those typo mistakes were corrected.

• Reference is missing in line 341

We apologize for this mistake. In fact, we were discussing and referring to our current results and not another study. Thus, we have removed the mention “ref” and we adjust the text accordingly.

The statement now is: “*In contrast to the decreased expression levels of the four genes and proteins in the PBMCs of a small set of subjects, no differences were observed in the DEFA1 and ANX3 plasma levels when analyzing a larger cohort of subjects in the present study*.”

• The statements from line 354 and line 360 should be paraphrased for clarity.

As suggested by the reviewer, this section was rephrased for clarity in our revised manuscript. Now it reads:

“Indeed, DEFA1 levels were similar between patients with middle-stage CHF (class I-II) and healthy controls but were significantly higher among patients with CHF in advanced stages (class III-IV) [29]. In addition, in the present report, no differences were observed in DEFA1 levels among patients with and without known CVDs. Circulating levels of DEFA1 were; however, reported to be linked to the CAD severity [30]. With regard to ANX3, little is known about its level in plasma when compared with other members of annexin family such as ANX1 [44]. Similarly, we did not observe a significant change in MMP9 levels in patients with CVD. Increased circulating levels of MMP9, however, have been reported in patients with ACSs [45] and peripheral arterial disease [46].”

**Reviewer #3:** The authors have taken a topic on cardio-metabolic diseases and using a shotgun proteomic approach, have shown novel proteins that are up and down-regulated in patients with CVD. This has been accompanied with their STRING network analysis which shows interlinks between the molecules and may present with proposed pathways. The identification of PR3, a neutrophil serine protease was up-regulated in CVD patients and presents itself as a novel marker in patients with CVD with diabetes and obesity.

We thank the reviewer for the very positive feedback on our manuscript.

We have further improved our manuscript throughout the suggestions from the other reviewers.

6. PLOS authors have the option to publish the peer review history of their article ([what does this mean?](https://journals.plos.org/plosone/s/editorial-and-peer-review-process#loc-peer-review-history)). If published, this will include your full peer review and any attached files.

If you choose “no”, your identity will remain anonymous but your review may still be made public.

**Do you want your identity to be public for this peer review?** For information about this choice, including consent withdrawal, please see our [Privacy Policy](https://www.plos.org/privacy-policy).
Reviewer #1: No
Reviewer #2: No
Reviewer #3: No

[NOTE: If reviewer comments were submitted as an attachment file, they will be attached to this email and accessible via the submission site. Please log into your account, locate the manuscript record, and check for the action link "View Attachments". If this link does not appear, there are no attachment files to be viewed.]

While revising your submission, please upload your figure files to the Preflight Analysis and Conversion Engine (PACE) digital diagnostic tool, <https://pacev2.apexcovantage.com/>. PACE helps ensure that figures meet PLOS requirements. To use PACE, you must first register as a user. Registration is free. Then, login and navigate to the UPLOAD tab, where you will find detailed instructions on how to use the tool. If you encounter any issues or have any questions when using PACE, please email us at [figures@plos.org](mailto:figures@plos.org). Please note that Supporting Information files do not need this step.
